# Effect of shading and light recovery on the growth, leaf structure, and photosynthetic performance of soybean in a maize-soybean relay-strip intercropping system

**DOI:** 10.1371/journal.pone.0198159

**Published:** 2018-05-31

**Authors:** Yuanfang Fan, Junxu Chen, Yajiao Cheng, Muhammad Ali Raza, Xiaoling Wu, Zhonglin Wang, Qinlin Liu, Rui Wang, Xiaochun Wang, Taiwen Yong, Weiguo Liu, Jiang Liu, Junbo Du, Kai Shu, Wenyu Yang, Feng Yang

**Affiliations:** 1 College of Agronomy, Sichuan Agricultural University, Chengdu, P.R. China; 2 Sichuan Engineering Research Center for Crop Strip Intercropping System, Chengdu, P.R. China; 3 Key Laboratory of Crop Ecophysiology and Farming System in Southwest, Ministry of Agriculture, Chengdu, P.R. China; Estacion Experimental del Zaidin, SPAIN

## Abstract

Intercropping is an important agronomic practice adopted to increase crop production and resource efficiency in areas with intensive agricultural production. Two sequential field trials were conducted in 2015–2016 to investigate the effect of shading on the morphological features, leaf structure, and photosynthetic characteristics of soybean in a maize-soybean relay-strip intercropping system. Three treatments were designed on the basis of different row configurations A1 (“50 cm + 50 cm” one row of maize and one row of soybean with a 50 cm spacing between the rows), A2 (“160 cm + 40 cm” two rows of maize by wide-narrow row planting, where two rows of soybean were planted in the wide rows with a width of 40 cm, and with 60 cm row spacing was used between the maize and soybean rows), and CK (sole cropping of soybean, with 70 cm rows spacing). Results showed that the photosynthetically active radiation transmittances of soybean canopy at V5 stage under A2 treatment (31.1%) were considerably higher than those under A1 (8.7%) treatment, and the red-to-far-red ratio was reduced significantly under A1 (0.7) and A2 (1.0) treatments compared with those under CK (1.2). By contrast with CK, stem diameter, total aboveground biomass, chlorophyll content and net photosynthetic rate decreased significantly except plant height under A1 and A2. The thickness of palisade tissue and spongy tissue of soybean leaf under A1 and A2 were significantly reduced at V5 stage compared with CK. The leaf thicknesses under A1 and A2 were lower than those in CK by 39.5% and 18.2%, respectively. At the R1 stage of soybean (after maize harvest), the soybean plant height, stem biomass, leaf biomass and petiole biomass under A1 and A2 treatments were still significantly lower than those under CK, but no significant differences were observed in Chl a/b, *P*_*n*_, epidermis thickness and spongy tissue thickness of soybean leaves in A2 compared with CK. In addition, the soybean yields (g plant^-1^) under A1 and A2 were 54.69% and 16.83% lower than those in CK, respectively. These findings suggested that soybean plants can regulate its morphological characteristics and leaf anatomical structures under different light environments.

## Introduction

Intercropping is an important cropping system extensively used worldwide for food and dietary fiber purposes. In intercropping system, at least two crops are grown on the same land for a specific period of time with alternating strips, which partially overlap in the growth period[1]. For instance, maize-soybean relay-strip intercropping is widely adopted in southwestern parts of China, where maize is sown in April and harvested in August, while soybean is sown in June and harvested in October[2]. The advantages of this system include the effective and efficient utilization of farmland resources[3] and low incidences of diseases, pests, and weed damage[4,5]. This system also increases the economic benefits compared with that of sole cropping of soybean[6,7]. The success of this approach is attributed to the efficient utilization of water and light, thereby increasing crop yield and improving the biodiversity and ecological services[8,9].

Cereals with legumes are well-known options in intercropping systems, and the maize-soybean intercropping system is a major intercropping pattern of cereal and legume in China[6]. Although both crop species compete for nutrients, especially nitrogen, because they require nitrogen for their growth, this competition forces legumes to fix nitrogen from the atmosphere and increase soil nutrients[10]. Such competition for soil nitrogen is reduced, and maize consequently absorbs more nitrogen from the soil than soybean[11]. This system has been widely used in developing countries[12] to improve the crop productivity[13]. The maize-soybean intercropping pattern has also been developed rapidly because it can effectively enhance soybean yield and plantation area and ensure maize production as well; for example, the plantation area in southwest of China is 6.67 million hectares[14]. The average seed yield of maize and soybean from this system are 7,000 kg and 1,300 kg ha^−1^ of maize and soybean, respectively[15,6].

Light directly affects the crop growth and yield potential. Co-growth period is essential for soybean growth in a maize-soybean relay-strip intercropping system because tall crops (maize) absorb major part of the light, whereas shorter crops (soybean) receive low amounts of light for photosynthesis and suffer shading from taller crops[16,7]. In this system, shade from maize severely affects the soybean growth and development; light intensity and light spectrum are also changed in maize-soybean relay strip intercropping systems[17]. Changes in irradiance influence the plant growth, morphology, and anatomy[18], and negatively affected the plant physiology and cellular biochemistry[19], and reduces the soybean leaf size by controlling cell proliferation[20]. Previous studies have focused on the leaf structure and functions of sorghum (*Sorghum bicolor* (*L*.) *Moench*), jasmine (*Jasminum sambac Ait*.), and peach (*Amygdalus persica L*.) under low light conditions [21,22,18]. In past reports it has been reported that the shading condition significantly changed the morphological characteristics, biomass, and physiological response of soybean seedlings [23,14]. Several studies have also provided insights into the leaf anatomical features of various plants under shading conditions [24,21,4].

However, no study has been conducted on the leaf anatomy and photosynthetic activity of soybean in a maize-soybean relay-strip intercropping system where soybean is first placed in a shaded environment in the vegetative growth period and then regains normal light after the maize harvest at reproductive period. These changed light conditions remarkably influences the yield potential and seed quality of soybean crop. The responses of soybean to shading and light recovery should be investigated for better management of this system. This study provides a new perspective to elucidate the effects of shading and light recovery on soybean morphology, leaf structure, and photosynthetic characteristics in maize-soybean relay-strip intercropping system. This study aims (1) to compare the properties of photosynthetically active radiation (PAR) and light quality on soybean canopy for A1 and A2 models in the relay-strip intercropping and sole cropping of soybean; (2) to investigate the changes in morphological characteristics, leaf structure, photosynthetic characteristics, and yield of soybean in response to shading and light recovery; and (3) to examine the effects of different treatments on the yield and yield components of soybean in relay-strip intercropping.

## Materials and methods

### Ethics statement

No specific permits were required for the described field studies. All experiments were performed according to institutional guidelines of Sichuan Agricultural University, China.

#### Experiment design

**Fig 1:**
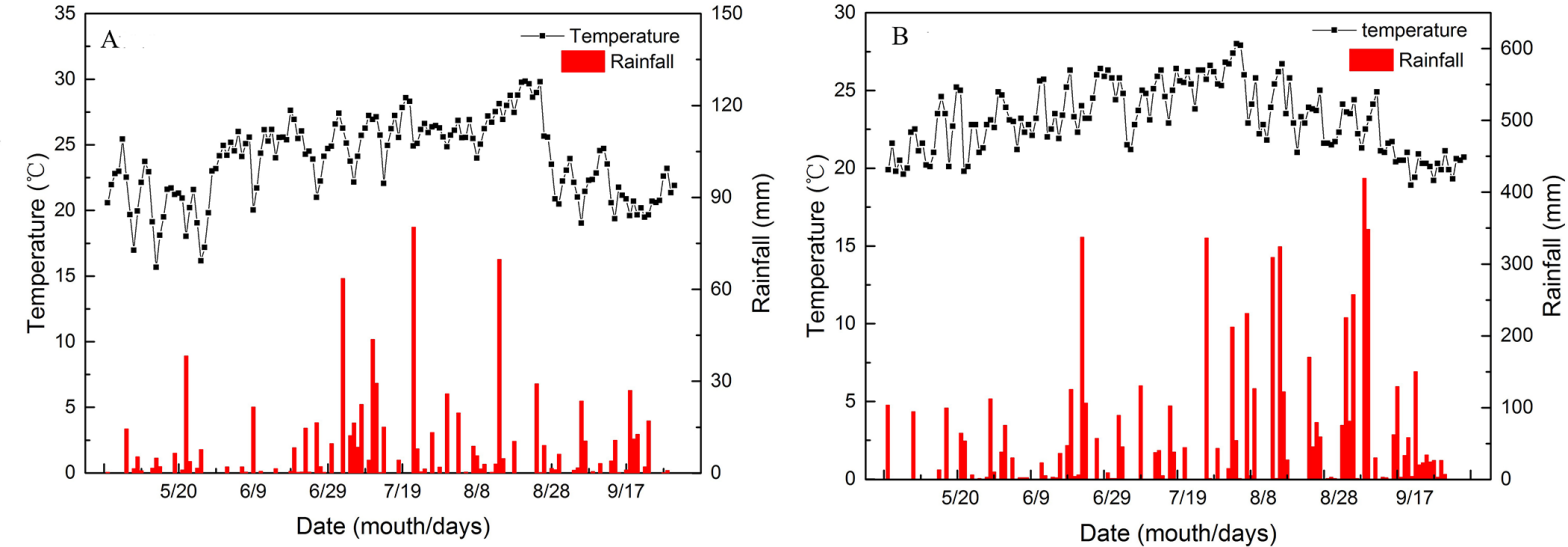
Air temperature and rainfall in the experimental site from May to September (during the sowing and harvesting of soybean) in 2015 and 2016. A: year 2015; B: year 2016. Data were obtained from Wenjiang Weather Bureau, Sichuan, China.

Field experiments were conducted in 2015 and 2016 at the Teaching and Experimental Farm of Sichuan Agricultural University in Wenjiang District, Sichuan, China (30° 71′N, 103° 86′E). The field climate of the experimental area was subtropical humid, with an annual average temperature of 16°C, an average annual rainfall of 865.9 mm, 991.1 sunshine hours, and an annual average frost-free period of 282 days. The air temperature and rainfall of the experimental site from May to September (from sowing to harvesting of soybean) in 2015 and 2016 are shown in Fig 1. We collected the weather data from Wenjiang Weather Bureau, Sichuan, China. The soil was aquod with a pH of 6.61.

**Fig 2:**
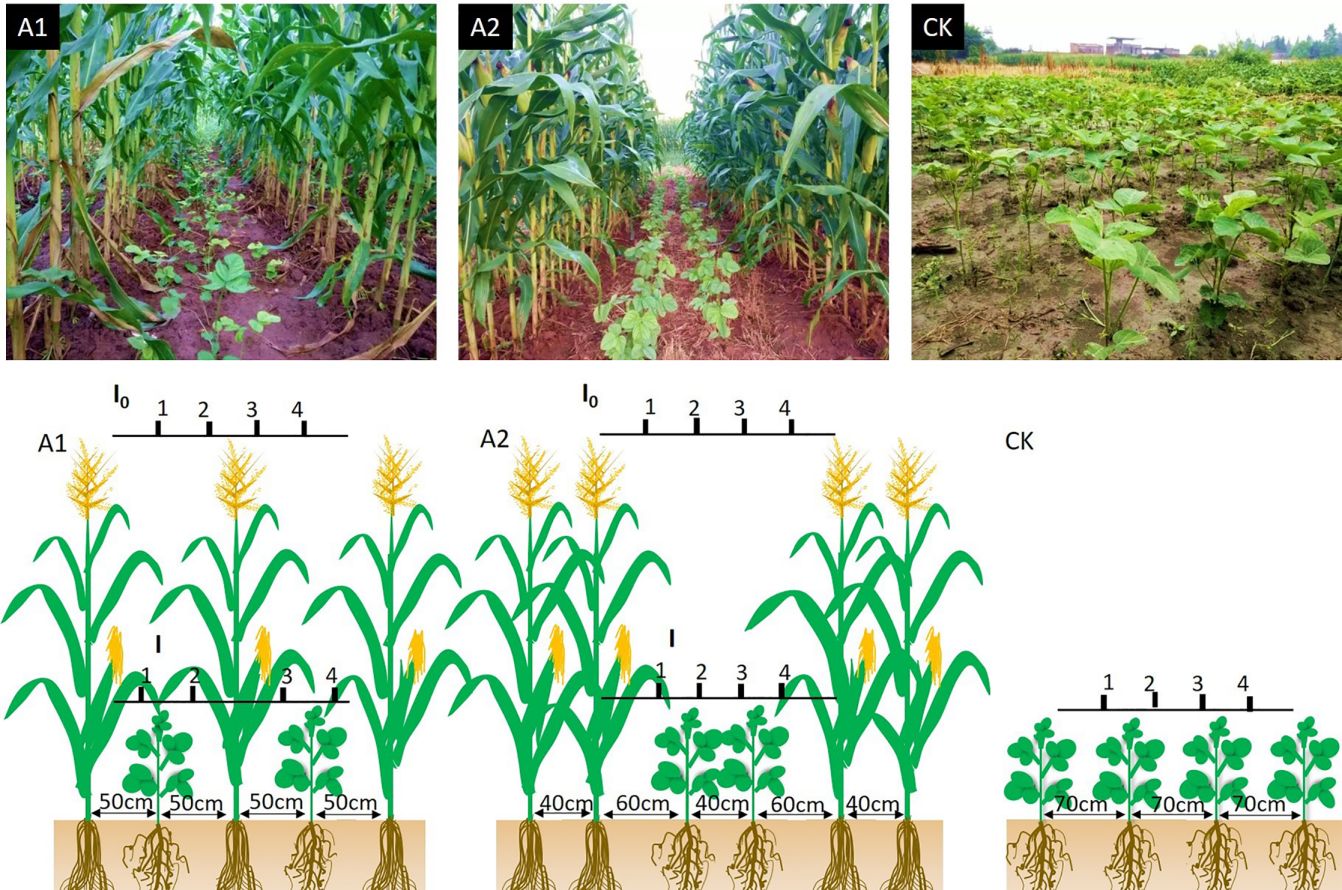
Maize-soybean relay-strip intercropping system and sole cropping system of soybean. A1: “50 cm + 50 cm” one row of maize and one row of soybean; A2: “160 cm + 40 cm” two rows of maize with wide-narrow row planting, where two rows of soybeans were planted in wide row with 40 cm; and with 60 cm row spacing between maize and soybean. CK: row spacing of soybeans in the sole cropping system was 70 cm.

Three treatments (Fig 2) were used in this study, namely, A1 (“50 cm + 50 cm” one row of maize and one row of soybean; A1 treatment is the traditional row intercropping planting patterns), A2 (“160 cm + 40 cm” two rows of soybean and two rows of maize with wide-narrow row planting, where soybeans were planted in two rows that are 40 cm in width; A2 treatment is the new planting pattern could increase peasant’s income), and CK (sole cropping of soybean). Each treatment was replicated three times. The plant-to-plant distances for intercropped maize and soybean were 16.7 and 10 cm, respectively. The row to row and plant to plant distance for sole cropping system of soybean were 70 and 14.3 cm, respectively. The soybean and maize plant densities were 100 000 plant·ha^-1^ and 60 000 plant·ha^-1^ in intercropping, respectively. The soybean plant density in sole cropping was consistent with that in intercropping system. The maize cultivar ‘Chuandan418’ and the soybean cultivar ‘Nandou12’ were used in this experiment. Maize was sown on March 27, 2015, and March 29, 2016, and soybean was sown on June 17, 2015, and June 19, 2016. Maize was harvested on August 10, 2015, and August 13, 2016, and soybean was harvested on October 21, 2015, and October 22, 2016. All of the plots were treated with basal fertilizer application: basal N at 37.5 kg ha^−1^ as urea, phosphorus at 600 kg ha^−1^ as calcium superphosphate, and potassium at 150 kg ha^−1^ as potassium chloride was applied to all treatments at the time of sowing. At the seedling, jointing, and bell-mouthed stages of maize, the second dose of N was applied at 75 kg ha^−1^, 150kg ha^-1^ as urea, and 750 kg ha^−1^ of ammonium bicarbonate was applied as fertilizer. At the time of soybean sowing, N at 75 kg ha^−1^ as urea, P at 600 kg ha^−1^ as calcium superphosphate, and K at 60 kg ha^−1^ as potassium chloride sulfate were applied, and at flowering stage of soybean, the second dose of N was applied at 75 kg ha^−1^ as urea.

#### Measurement of PAR and spectral irradiance

PAR under different treatments of intercropping systems was measured to characterize the light environment at different positions at the top of soybean canopy by using quantum sensors (Li-1400; America, LI-COR). These sensors were placed horizontally 5 cm above the soybean canopy (Fig 2). All of the measurements were made between 11:00 and 13:00 on cloudless days at V5 and R1 stages of soybean growth[25]. PAR transmittance was calculated by using the following formula:

$$PAR\;transmittance\;(\%)=\frac{I}{I_{0}}\times 100\%$$ (1)

where *I* is the PAR at the top of the soybean canopy and *I*_0_ is the PAR at the top of the maize canopy.

After each PAR measurement was conducted, the spectral irradiance of the soybean canopy was determined with a fiber optic spectrometer (Ava Spec-2048; Avantes, Netherlands) at the corresponding measuring point. Spectral irradiance was originally measured at wavelengths ranging from 350 nm to 1,000 nm, and the probe of the spectrometer was directed upward at the measuring point.

#### Measurement of plant growth

Five uniform plants were sampled from each plot for morphological analysis. Plant height and stem diameter were measured using a ruler and a Vernier caliper, respectively. All of the aboveground parts of the soybean plants were then divided into leaves, petioles, and stems, dried in an oven at 105°C for 1 h, and dried to a constant weight at 75°C to determine the total biomass of the leaves, petioles, and stems.

#### Measurement of photosynthetic pigment content

Three fully expanded leaves of soybean plants in each treatment were collected at the fifth trifoliolate (V5) and beginning bloom (R1) stages. Chlorophyll and carotenoids were extracted from the middle lobules of the third leaf (from top to bottom) of soybean plants, and two leaf discs (1.130 cm^2^) were cut from the middle part of each middle lobules by a puncher (1.2 diameter), and dipped in 10 ml of 80% aqueous acetone solution and placed in the dark for 24 h at room temperature[26]. The extract was then measured at wavelengths of 663, 646, and 470 nm by using a spectrophotometer (DU-730; America Beck Man Coulter). The photosynthetic pigment was calculated by using the following formula[27]:

$$C_{a}(mg∙L^{-1})=12.21A_{663}-2.81A_{646}$$ (2)

$$C_{b}(mg∙L^{-1})=20.13A_{646}-5.03A_{663}$$ (3)

$$C_{car}(mg∙L^{-1})=(1000A_{470}-3.27C_{a}-104C_{b})∙229^{-1}$$ (4)

$$Pigment\;content\;(mg∙{dm}^{-2})=C∙V∙S^{-1}$$ (5)

C_a,_ C_b,_ and C_car_ were the Chla, Chlb and carotenoid concentration, respectively. The photosynthetic pigment content can be calculated by formula (5). Where C in formula 5 is the photosynthetic pigment concentration, V is the pigment extract volume and S is the leaf discs area.

#### Measurement of photosynthesis

Photosynthetic measurements were carried out by using portable photosynthesis equipment (LI-6400, Li-COR, USA) with steady light intensity (1,000 μmol m^−2^ s^−1^) at V5 and R1 stages of soybean growth from 10:00 to 11:00 at a CO_2_ concentration of 400 µmol mol^−1^.

#### Measurement of leaf anatomical features

At V5 and R1 stages of soybean growth, the middle leaflet of the three latest fully expanded leaves was sampled by using a pair of surgical scissors, and the leaf segments (5 × 10 mm) without veins were fixed with a formaldehyde-glacial acetic acid-alcohol (FAA) solution (38% formaldehyde/glacial acetic acid/70% alcohol, 5:5:90, V/V) at 4°C[28]. The fixed segments were then dehydrated in a graded alcohol and *n*-butyl alcohol series, embedded in paraffin, and cut by a rotary microtome (RM2235, Leica Microsystems Ltd., Germany) at a thickness of 10 μm. Light microscopy (Nikon Eclipse 50i; Japan) was performed by using a 10 μm thick transverse section of the leaf stained with fast green and the counterstain safranin. The thicknesses of leaf, adaxial and abaxial epidermis, palisade, and spongy mesophyll tissues were obtained using Image J 1.42q.

#### Measure of soybean yield

Five consecutive plants were used for calculating the yield and yield components of soybean at R7 stage. The number of branches, pods per plant, grains per plant, hundred grain weight, and yield per plant of soybean were determined.

#### Statistical analysis

Microsoft Excel 2013 was used for data-calculated analysis. SPSS version 19.0 was utilized to compare data through one-way ANOVA and test the differences among A1, A2, and CK. Origin Pro 9.1 was employed to draw all the figures.

## Results

### Light environment of soybean canopy

**Fig 3:**
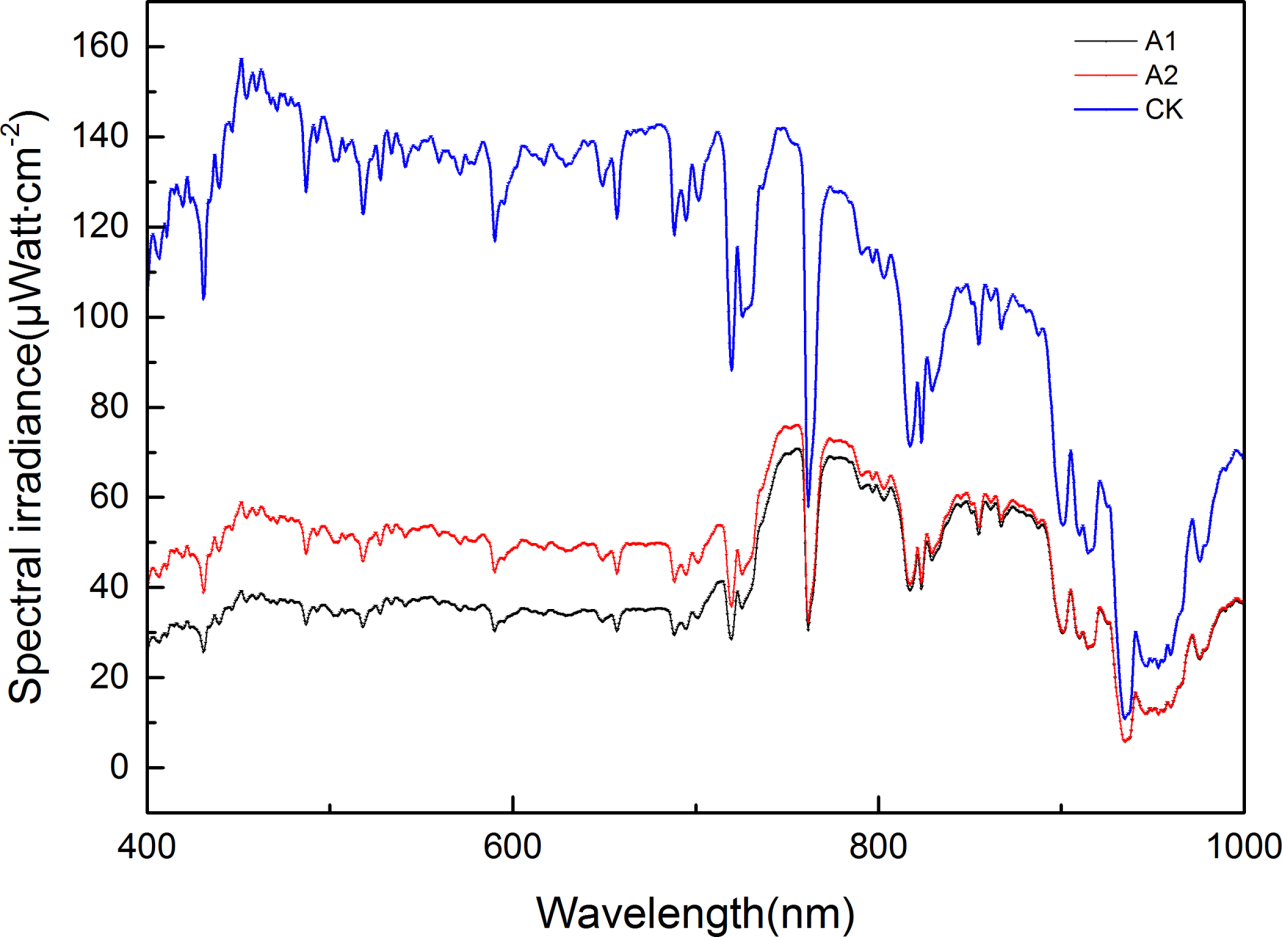
Changes in the spectral radiance of soybean canopy in maize-soybean relay-strip intercropping system and sole cropping system.

The light quality (red–far red [R/FR] ratio) and quantity (PAR) changed significantly in all treatments at the top of the soybean canopy in A1 and A2 compared with those of the sole cropping system (Fig 3 and Table 1). Compared with CK, the PARs in A1 and A2 decreased by 91.2% and 66.8%, respectively, and their transmittances were 8.7% and 31.1% of CK, respectively (average values in 2015 and 2016). The spectral irradiance at the top of the soybean canopy significantly differed among A1, A2, and CK in the V5 stage of soybean growth (Fig 3). The spectral irradiance of soybean canopy in the relay-strip intercropping system was lower than that in the sole cropping system. Compared with CK, the R/FR ratios under A1, A2, decreased by 0.7, 1.0, respectively.

**Table 1:** Light distribution of soybean canopy under A1, A2, and CK treatments in the V5 stage of soybean growth in 2015 and 2016.

Year	2015		2016	
Treatments	PAR (mol·m^−2^·s^−1^)	Transmittance (%)	PAR (mol·m^−2^·s^−1^)	Transmittance (%)
A1	174.67±3.06c	9.07±0.17c	157.67±0.58c	8.73±0.14c
A2	640.00±5.29b	34.26±1.10b	537.67±2.89b	28.04±1.51b
CK	1922.63±1.76a	100.00±0.00a	1845.67±17.90a	100.00±0.00a

Different lowercase letters in the same column are significantly different at 0.05 probability level by Duncan’s multiple range test. Data are means ± SD of three replicates.

### Plant growth

**Fig 4:**
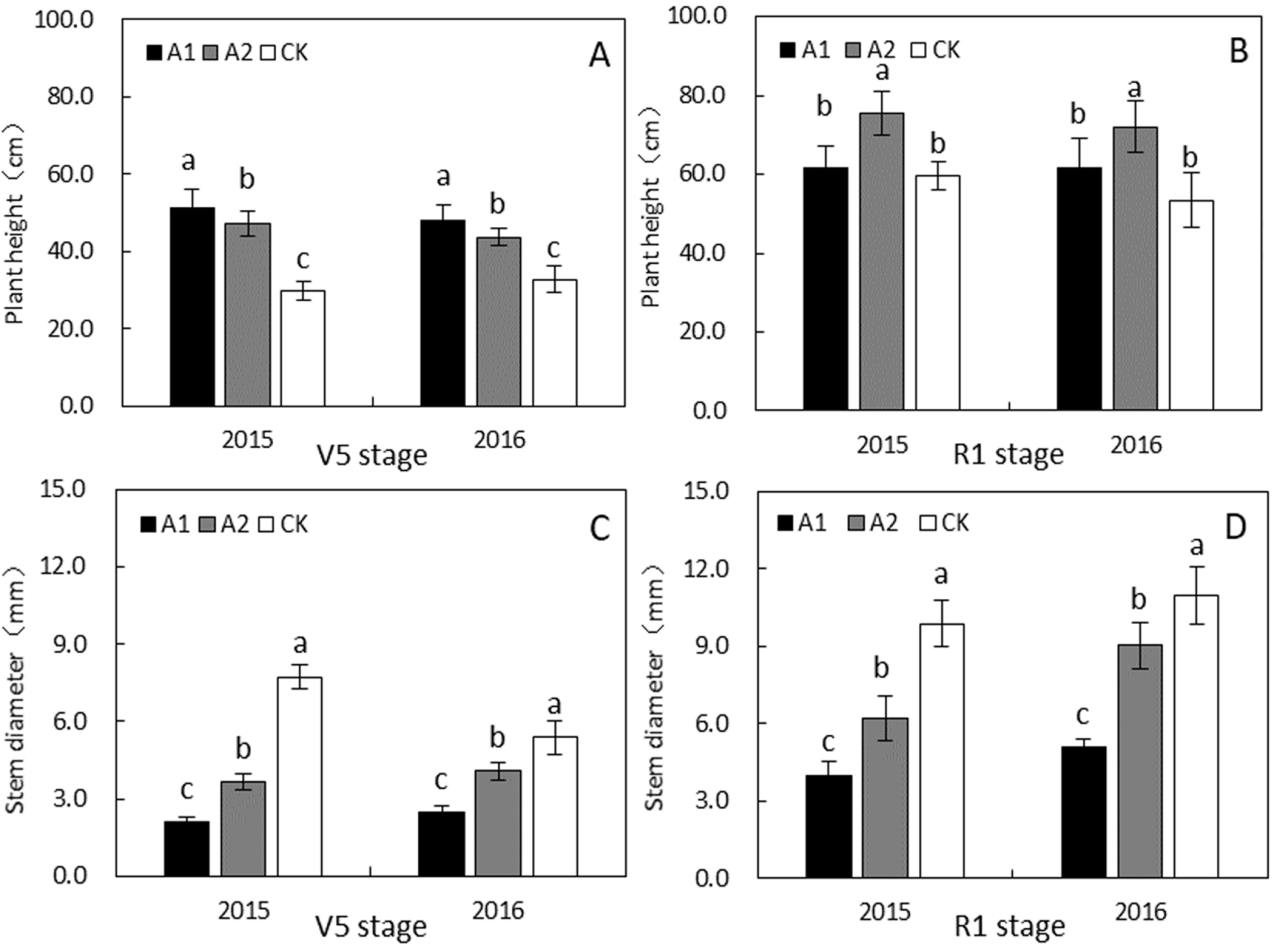
Changes in the morphological characteristics of soybean in the maize-soybean relay-strip intercropping system and sole cropping system in 2015 and 2016. Bars represented by different small letters within each group in the same growth stage are significantly different at the 0.05 probability level. Values are means ± SD of three replicates.

Plant height and stem mass ratio increased significantly (Fig 4A and Table 2), whereas the stem diameter (Fig 4C), stem biomass, leaf biomass, petiole biomass, leaf mass ratio, and petiole mass ratio decreased significantly at V5 stage of soybean growth under A1 compared with sole cropping system (Table 2). At R1 stage, the plant height in A1 did not significantly differ from that in CK. By contrast, the plant height under A2 at the R1 stage was higher than those under A1 and CK (Fig 4B). Compared with CK, the mean stem diameter and the aboveground accumulation of the biomass of soybean plants decreased under A1 and A2 at V5 and R1 stages of soybean growth (Fig 4D and Table 2).

**Table 2:** Changes in the aboveground biomass accumulation and distribution of soybean in the maize-soybean relay-strip intercropping system and sole cropping system.

		V5 stage (shading period)			R1 stage (Light recovery period)		
Year	Index	A1	A2	CK	A1	A2	CK
	Stem biomass (g)	0.372±0.04b	1.228±0.28a	1.281±0.20a	2.257±0.10c	7.356±0.53b	12.307±1.91a
	Leaf biomass (g)	0.366±0.09c	1.544±0.39b	2.362±0.28a	1.894±0.32c	7.210±0.80b	14.256±1.44a
	Petiole biomass (g)	0.074±0.02c	0.405±0.11b	0.629±0.05a	0.469±0.09c	2.212±0.53b	5.282±0.39a
2015	Above ground biomass(g)	0.811±0.14c	3.177±0.78b	4.271±0.49a	4.62±0.48c	16.78±1.80b	31.85±2.31a
	Stem biomass ratio (%)	46.22±3.5a	38.77±1.04b	29.91±1.68c	49.07±3.15a	43.96±1.92ab	38.58±4.66b
	Leaf biomass ratio (%)	44.75±3.34b	48.54±0.98b	55.23±1.74a	40.81±2.79a	42.96±0.17a	44.78±3.29a
	Petiole biomass ratio (%)	9.03±1.11c	12.69±0.30b	14.80±1.13a	10.12±1.25b	13.08±1.78ab	16.63±1.55a
	Stem biomass (g)	0.346±0.09b	1.288±0.14a	1.436±0.20a	3.57±0.09c	7.70±0.34b	14.04±0.44a
	Leaf biomass (g)	0.395±0.08c	1.566±0.15b	2.77±0.26a	2.70±0.19c	7.02±0.18b	17.00±0.03a
	Petiole biomass (g)	0.054±0.01c	0.367±0.07b	0.579±0.06a	0.95±0.03c	2.56±0.46b	5.43±0.26a
2016	Above ground biomass(g)	0.796±0.17c	3.261±0.33b	4.854±0.54a	7.21±0.30c	17.28±0.39b	36.47±0.69a
	Stem biomass ratio (%)	43.15±2.93a	39.61±3.61a	29.55±1.46b	49.51±0.85a	44.56±2.17b	38.49±0.51c
	Leaf biomass ratio (%)	49.83±2.52b	48.25±5.49b	57.27±2.22a	37.36±1.02c	40.67±1.58b	46.63±0.87a
	Petiole biomass ratio (%)	7.60±1.18b	9.41±1.80b	11.95±0.32a	13.13±0.20a	14.77±2.36b	14.88±0.45a

Different lowercase letters in the same line are significantly different at 0.05 probability level by Duncan’s multiple range test. Data are means ± SD of three replicates.

### Photosynthetic pigment content

The chlorophyll content of the functional leaves is shown in Table 3. The Chl a and carotenoid (Car) contents under A1 and A2 treatments decreased significantly comparing to those under CK treatment in V5 and R1 stages of soybean, whereas no significant difference for Chl a/b was found between A2 and CK treatment at R1 stage. Compared with CK, the Chl a and Chl b contents decreased considerably under A1 and A2 by 50.2%, 27.9%, and 46.4%, 13.7% at V5 stage of soybean growth. The Chl a content was significantly affected by shading in the maize-soybean relay-strip intercropping system at V5 and R1 stages of soybean growth.

**Table 3:** Soybean leaf photosynthetic pigments in maize-soybean relay-strip intercropping system and sole cropping system (mg·dm^−2^).

Years	Growth stage	Treatment	Chl a	Chl b	Chl (a+b)	Car	Chl a / b
2015	V5 stage	A1	1.820±0.060c	0.463±0.022c	2.283±0.058c	0.429±0.025c	3.937±0.251ab
		A2	2.843±0.135b	0.797±0.014b	3.640±0.136b	0.658±0.031b	3.567±0.179b
		CK	4.410±0.285a	1.054±0.094a	5.464±0.378a	1.086±0.070a	4.189±0.129a
	R1 stage	A1	2.741±0.115b	0.764±0.065b	3.505±0.102b	0.679±0.013b	3.611±0.409a
		A2	3.105±0.273b	0.855±0.50b	3.959±0.291b	0.714±0.060b	3.637±0.311a
		CK	4.291±0.180a	1.041±0.077a	5.332±0.255a	1.113±0.105a	4.129±0.150a
2016	V5 stage	A1	2.228±0.092 c	0.547±0.025 b	2.776±0.117 c	0.516±0.038 c	4.071±0.032 b
		A2	3.044±0.116 b	0.839±0.023 a	3.883±0.138 b	0.619±0.025 b	3.626±0.040 c
		CK	3.817±0.060 a	0.866±0.037 a	4.683±0.090 a	0.850±0.009 a	4.413±0.150 a
	R1 stage	A1	3.030±0.273 c	0.769±0.080 b	3.799±0.352 c	0.722±0.064 b	3.947±0.077 b
		A2	3.488±0.199 b	0.828±0.064 b	4.316±0.252 b	0.798±0.050 b	4.224±0.208 a
		CK	4.027±0.164 a	0.931±0.044 a	4.958±0.189 a	0.921±0.084 a	4.328±0.189 a

Different lowercase letters in the same column are significantly different at 0.05 probability level by Duncan’s multiple range test. Data are means ± SD of three replicates.

### Photosynthetic characteristics

The photosynthetic characteristics, including net photosynthetic rate (*P*_*n*_), stomatal conductance (*G*_*s*_), intercellular CO_2_ concentration (*C*_*i*_), and transpiration rate (*T*_*r*_), of functional leaves were measured to explain the soybean leaf responses to different intercropping systems. Compared with sole cropping of soybean, *P*_*n*_, *G*_*s*_, and *T*_*r*_ decreased significantly under in the maize-soybean relay-strip intercropping patterns, but *C*_*i*_ increased at the V5 stage of soybean growth (Table 4). *P*_*n*_ of A1 and A2 respectively decreased by 64.7% and 35.3% compared with those of the sole cropping system. In addition, the photosynthetic characteristic parameters of soybean plants at R1 stage followed a pattern similar to that at the V5 stage, but the *P*_*n*_ under maize-soybean relay-strip intercropping system increased, and *P*_*n*_ of the functional leaves under A2 did not significantly differ from that of the sole cropping system (Table 4).

**Table 4:** Soybean leaf photosynthetic characteristics in maize-soybean relay-strip intercropping system and sole cropping system.

Year	Growth stage	Treatment	*P*_n_ (μmol CO_2_ m^-2^ s^-1^)	*G*_s_ (mol H_2_O m^-2^ s^-1^)	*C*_i_ (μmol CO_2_ m^-2^ s^-1^)	*T*_r_ (mmol H_2_O m^-2^ s^-1^)
2015	V5 stage	A1	5.210±0.018c	0.074±0.001c	237.238±0.477a	2.934±0.011c
		A2	13.185±1.213b	0.172±0.003b	219.273±0.486b	5.838±0.161b
		CK	17.967±0.137a	0.163±0.001a	162.620±0.087c	6.280±0.061a
	R1 stage	A1	11.334±0.601b	0.172±0.001c	262.881±0.959c	2.317±0.003b
		A2	13.146±0.577a	0.290±0.001b	277.701±1.648b	3.378±0.004a
		CK	14.220±0.559a	0.314±0.090a	294.623±0.027a	3.550±0.182a
2016	V5 stage	A1	6.255±0.494 c	0.659±0.032 c	342.891±0.554 a	3.508±0.144 c
		A2	11.284±0.439 b	0.900±0.057 b	335.127±7.861 a	4.945±0.303 b
		CK	17.823±0.205 a	1.157±0.010 a	321.227±2.323 b	6.679±0.184 a
	R1 stage	A1	11.327±1.552b	0.181±0.034 c	271.001±6.539b	2.425±0.267c
		A2	13.683±0.639a	0.301±0.078 b	290.742±24.929a	3.336±0.427b
		CK	14.721±0.693a	0.379±0.089 a	300.997±17.650a	4.030±0.665a

*P*_*n*_: Photosynthetic rate, *G*_*s*_: Conductance to H_2_O, *C*_*i*_: Intercellular CO_2_ concentration, *T*_*r*_: Transpiration rate. Different lowercase letters in the same column are significantly different at 0.05 probability level by Duncan’s multiple range test. Data are means ± SD of three replicates.

### Leaf anatomical structure of soybean

**Fig 5:**
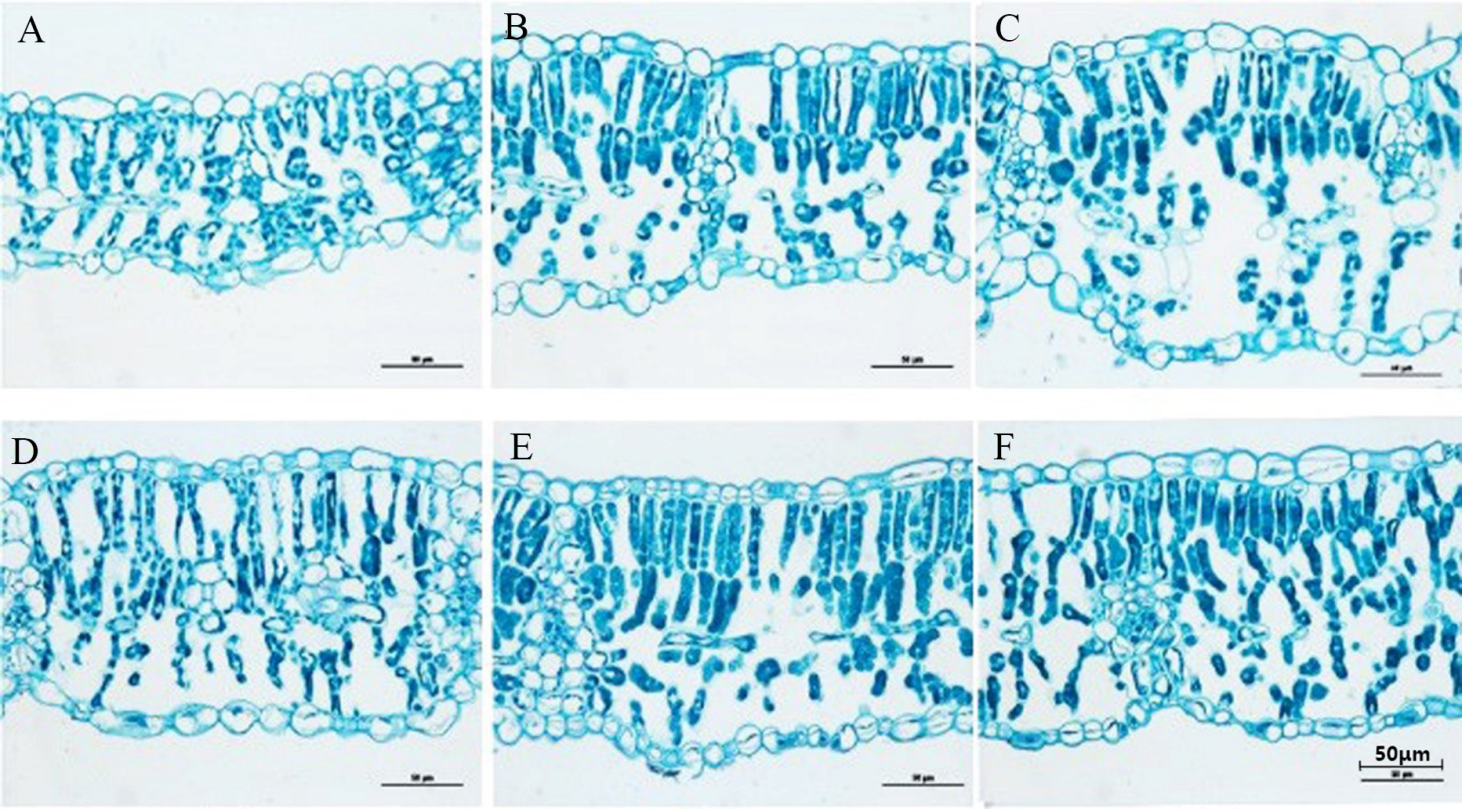
Changes in the leaf anatomical structure of soybean in the maize-soybean relay-strip intercropping system and the sole cropping system. A, B and C: A1, A2 and CK (V5 stage), respectively; D, E and F: A1, A2 and CK (R1 stage), respectively.

The soybean compound leaf was bifacial and differentiated into palisade and spongy tissues. Two layers of long columnar palisade tissue cells were under the upper epidermal cells, and many spongy tissues were between the palisade tissue and the lower epidermal cells. The effects of A1 and A2 treatments on the cross section of soybean leaves are illustrated in Fig 5. The leaves under A1 and A2 were 39.5% and 18.2% thinner than that under CK, respectively. Similarly, the thicknesses of the palisade and spongy tissues were significantly decreased by 54.6%, 21.7%, 42.3%, and 19.5%, respectively (Table 5). No significant difference in the thicknesses of the upper epidermal cells and the lower epidermal cells were found among all the treatments (Table 5). In the subsequent recovery period (R1 stage of soybean), the plant rebounded quickly after shading, and the thicknesses of the palisade tissue and leaves significantly decreased by 20.7% and 12.2% and by 14% and 7.3% under A1 and A2, respectively. The thicknesses of the epidermal cell and spongy tissues slightly differed (Table 5).

**Table 5:** Leaf anatomical structure of soybean in the maize-soybean relay-strip intercropping system and the sole cropping system.

Year	Growth stage	Treatment	Upper epidermis thickness (μm)	Lower epidermis thickness (μm)	Palisade tissue thickness (μm)	Sponge tissue thickness (μm)	Leaf thickness (μm)
2015	V5 stage	A1	12.87±0.41a	10.88±0.59a	30.06±3.00c	29.30±3.00c	91.01±3.23c
		A2	15.80±1.79a	13.95±1.43a	50.43±5.58b	36.39±5.58b	108.79±3.43b
		CK	13.93±1.10a	13.88±1.73a	63.95±3.52a	41.16±3.52a	131.36±4.02a
	R1 stage	A1	11.54±0.33a	10.54±1.85a	65.07±4.19b	29.17±1.30a	120.85±2.59b
		A2	11.93±0.58a	13.90±0.45a	72.62±4.25b	33.11±1.00a	131.06±5.42ab
		CK	10.61±0.66a	11.73±1.35a	82.67±0.45a	34.05±3.75a	142.35±0.80a
2016	V5 stage	A1	13.76±1.80a	12.38±1.70b	37.36±4.32c	27.69±3.66c	89.51±7.08c
		A2	14.48±1.40a	13.87±1.76ab	64.25±2.33b	47.53±8.92b	139.85±9.85b
		CK	13.56±1.20a	14.54±1.62a	83.0±6.56a	65.48±9.02a	172.98±11.85a
	R1 stage	A1	12.73±1.09a	12.00±0.60a	66.80±3.71a	47.66±2.09a	140.56±6.99b
		A2	11.44±0.54a	14.98±1.21a	66.64±4.42a	47.35±7.08a	149.07±2.46b
		CK	11.65±0.55a	13.26±1.97a	70.38±1.08a	61.14±8.59a	160.09±3.14a

Different lowercase letters in the same column are significantly different at 0.05 probability level by Duncan’s multiple range test. Data are means ± SD of three replicates.

### Yield and yield components of soybean

Yield is the result of the coordination of yield components, and this parameter is determined by various agronomic traits, such as height, pod number, and effective branching number. The results of yield and yield components are shown in Table 6.The number of branches, pods per plant, grains per plant, hundred grain weight, and yield per plant of soybean under A1 and A2 decreased considerably compared with those under CK. The yield per plant in A1 and A2 decreased by 54.69% and 16.83% in the maize-soybean relay-strip intercropping system.

**Table 6:** Soybean yield and component factors in maize–soybean relay-strip intercropping systems.

Year	Treatments	Number of branches	Pods per plant	Grains per plant	Hundred grain weight (g)	Yield per plant (g plant^−1^)
2015	A1	2.40±0.548b	43.00±4.637c	76.20±2.588c	22.47±0.257b	15.84±1.262c
	A2	3.80±1.483b	78.60±6.580b	112.40±7.829b	25.60±0.874a	28.85±4.523b
	CK	5.60±0.894a	102.60±9.813a	177.80±9.039a	25.92±0.691a	35.16±3.195a
2016	A1	3.00±0.63c	45.60±2.24c	69.40±3.38c	19.38±0.25b	13.45±1.44c
	A2	4.80±0.63b	66.80±1.33b	110.40±5.49b	22.54±0.34a	24.88±3.37b
	CK	7.00±1.10a	83.80±3.19a	129.00±4.20a	22.88±0.06a	29.52±2.28a

Different lowercase letters in the same column are significantly different at 0.05 probability level by Duncan’s multiple range test. Data are means ± SD of three replicates.

## Discussion

### Morphological responses of soybean to shading and light recovery

Soybean is a light-loving plant; it can changed their botanical characteristics to adapt different environmental conditions such as with shading or weak light [29]. In our study, the soybean plant height was increased under maize sowing relay strip intercropping system, whereas the stem diameter and aboveground biomass accumulation was decreased during the intergrowth period compared with that of the sole cropping system (Fig 4 and Table 2). Shading conditions under A1 and A2 probably promoted the stem elongation and inhibited the stem diameter growth to obtain high amounts of light[30].

The dry matter production center is moved from leaves to stems under shaded conditions. Shade-avoiding plants often exhibit increased stem and hypocotyl elongation rates at the expense of the leaves [31,28]. The stem diameter and aboveground biomass accumulation under A1 were the lowest among all the treatments (Fig 4 and Table 2). Similarly, previous studies reported that the negative effect of shading on soybean growth because close planting of maize causes severe shading and absorbed most part of the light under maize-soybean relay-strip intercropping system [32,14,17]. Therefore, the R/FR ratio at the soybean canopy was reduced, which subsequently induce the shade avoidance responses in soybean plants [33]. The plant height of soybean under A1 did not differ from that of CK after normal light was regained, and the stem diameter and aboveground biomass accumulation of soybean under A1 and A2 increased rapidly compared with those of CK (Fig 4 and Table 2), that is because of the marginal effect at soybean after the maize harvest. Our results show an outstanding potential for maize-soybean intercropping, especially under more marginal conditions, as previously reported in Guinea savanna [30].

### Photosynthetic responses of soybean to shading and light recovery

Light is a major factor that influences the plant growth and development; through photosynthesis, plants use sunlight to convert water and carbon dioxide into sugar, and photosynthetic pigments play a key role in the process of changing light energy to chemical energy [34,21]. In shaded environments, the measurement of leaf chlorophyll contents serves as an effective indicator for light absorption[35].

Several studies have claimed that Chl a and Chl b contents decrease as the shading density increases[36]. Conversely, other studies have argued that chlorophyll contents increase as shading density increases, especially Chl b content increases [23]. Our results demonstrated that the Chl a and Chl b contents were significantly decreased under shading conditions (Table 3), and this result was closely related to leaf thickness. The reduction of PAR at the top of soybean canopy under A1 and A2 led to a decrease the photosynthetic rate, stomatal conductance, and transpiration rate of soybean plants, but the intercellular CO_2_ concentration increased at V5 stage of soybean growth. Consequently, this indicated that the reduced net photosynthetic rate under shading conditions was not caused by stomatal effect[37].

The photosynthetic characteristics of the functional leaves at R1 stage were generally similar to those at V5 stage. However, the net photosynthetic rate under relay-strip intercropping system increased, and the non-significant differences were observed between A2 and CK (Table 4). The recovery of soybean leaf functions under A2 were higher than A1 after maize harvest. Therefore, this suggested the A2 treatment exhibited a higher photosynthetic capacity than A1 treatment in the relay-strip intercropping system.

### Leaf anatomical structure responses of soybean to shading and light recovery

Leaves are implicated in process of photosynthesis and the most vital plant part during long-term evolution [28]. Plants adjust their morphological structures and physiological function leaves to adapt different environmental conditions[38]. Our research indicated that the shading of maize in the relay-strip intercropping system induced changes in the leaf anatomy of soybean (Fig 5 and Table 5).

Soybean leaves are typically bifacial and asymmetrical[22]. In our study, the palisade thicknesses and spongy tissues in soybean leaves under A1 were more sensitive than those in other treatments (Fig 5A). The loose arrangement and large cell gap were determined in cells of the shade leaves, and the thicknesses of the palisade tissues and leaves significantly decreased under A1 and A2, it maybe because of the weakened cell division in vertical directions and the decreased cell growth rate and number of cell layers in palisade tissues[39]. These structural characteristics enhance the reflection and scattering of light in leaves and allow high amounts of energy to reach plants under limited light conditions [40]. In this study, the thicknesses of the lamina in A1 and A2 treatments were significantly decreased compared with that in CK (Fig 5 and Table 5). This result may be ascribed to the decrease in the thickness of palisade parenchyma. Thus, the changes in the thickness of palisade tissues are the main targets of the systemic regulation of leaf morphology and anatomy in soybean seedlings in the relay-strip intercropping system.

During the subsequent recovery (R1 stage of soybean growth) after maize harvest, the leaves of soybean rebounded quickly after they regained normal light. The thicknesses of the leaves, palisade tissues, and spongy tissues under A1 and A2 were well developed, and their differences were less evident than those of CK (Fig 5D and 5E and Table 5). The palisade tissue elongation increased the area of chloroplast channel through which CO_2_ enters, thereby increasing the leaf thickness and strengthening the photosynthetic ability under normal light conditions[41,42]. This phenomenon is one of the main causes of the increase in the photosynthetic rate under A1 and A2 in the light recovery period.

The photosynthetic capacity of soybean is closely related to its yield, and leaf photosynthesis is the material basis of its grain formation [42]. In the present experiment, the number of pods of single plants and the number of grains of single plants in A1 and A2 treatments significantly decreased, compared with CK (Table 6), and the yield and component factors of the A2 treatment performed better than A1 treatment.

## Conclusions

The shading of maize affected the morphological features, leaf anatomical structure, and photosynthetic characteristics of soybean, thereby increasing soybean plant height and decreasing the stem diameter, biomass of aboveground parts, chlorophyll content, and leaf thickness in the maize-soybean relay-strip intercropping system. The stem diameter, biomass of aboveground parts, Chla content, net photosynthetic rate, and leaf thickness of soybean increased rapidly because of the restoration of the illumination environment of soybean canopy after the maize was harvested, thereby providing the material and energy base for the photosynthetic compensatory growth of soybean in later periods. The morphological and photosynthetic characteristics of soybean under A1 and A2 were also different during shading and light recovery. The soybean leaf anatomical structure under A2 treatment has a good adaptability, and the palisade tissue and spongy parenchyma were further developed. Therefore, optimizing population allocation is conducive to the plasticity of soybean growth regulation and the coordinated high yield of maize and soybean in the maize-soybean relay-strip intercropping system.
